# Felsin in Dyspepsia

**Published:** 1903-07

**Authors:** 


### Felsin in Dyspepsia.

J. A. Clement, Baltimore, says he does not regard the word biliousness as a scientific expression, but as the laity know what it means, or think they do, it cannot easily be stricken from the vocabulary. He regards the new preparation, felsin, of the Viskolein Co., as a useful remedy in certain forms of dyspepsia, especially when associated with biliousness.

He reports results in four cases: In each one of these he used felsin and nothing else. He wished to test the value of the preparation, hence these cases show bona fide results from the use of the felsin.

Case I.—Mr. G., aged 40; occupation, patrolman. This patient is so situated that his meals are taken very irregularly and, as a rule, hurriedly. Natural result—dyspepsia. On January 18, 1903, called for prescription; complained of accumulation of much gas and rumbling in abdomen, pyrosis, bad taste in mouth, a sensation of "a rock in my stomach," bowels irregular and slight frontal headache. Gave felsin tablets, one after each meal and one at bedtime. February 1st, reported that he had received considerable relief. Continued the prescription. February 7, reported all symptoms cleared up with the exception of the constipation, which was improving. Ordered felsin, one tablet after meals, for an indefinite time.

Case II.—Mr. M., aged 53; no occupation. He belongs to that class of dyspeptics who are imprudent, both in eating and drinking. His main complaint was excessive flatulence, bilious headache and constipation. Gave felsin tablets every four hours for three days, then one tablet after each meal and one at bedtime. The flatulence disappeared in two days, the headache in three and the bowels are beginning to act as they should. The tablets acted in this case very nicely. From the patient's description, he had about run through the list of digestives.

Case No. 3.—Mrs. C., aged 65. For three months past has noticed that if she takes anything more for supper than a cup of tea and a cracker or two she would awaken next morning with headache and more or less nausea. Also reported chronic constipation. Ordered felsin tablets after each meal and on going to bed. The first two mornings after using the tablets awakened with headache, but no nausea. Has now been using the tablets two weeks and can eat a moderate meal at night with no bad results next day.

Case No. 4.—Mrs. D., aged 24. Four months advanced in first pregnancy; complains that everything she eats creates gas, which she finds it difficult to get rid of. Complaints of pregnant women, of course, must be taken with a grain of salt, but I prescribed felsin, one tablet after meals. After eight days' treatment she reported that she had no more difficulty with the gas and that now she can't get enough to eat. Evidently felsin in this case relieved the gas and stimulated the appetite.

